# Perinatal palliative care: how to approach antenatal counselling

**DOI:** 10.1136/archdischild-2025-329875

**Published:** 2026-04-15

**Authors:** Sophie Bertaud, Paula-Ann Bailey, Sara Balmforth, Georgina Brightley, Sharon English, Alison Pearson, Katrina Williams, Association of Paediatric Palliative Medicine (APPM) Perinatal Special Interest Group, Lucinda Perkins, Michelle Hills, Gemma Holder, Christine Mott, Ross Smith, Hannah Linford, AK Anderson, Surabhi Nanda, Satyajit Ray, Fauzia Paize, Judith McKibben, Anne-Marie Harris

**Affiliations:** 1 Ethox Centre, Nuffield Department of Population Health, University of Oxford, Oxford, UK; 2 Louis Dundas Centre for Children’s Palliative Care, Great Ormond Street Hospital for Children, London, UK; 3 Keech Hospice, Luton, UK; 4 Regional Advice and Facilitation Team, East of England Children’s Palliative Care Service, Addenbrookes Hospital, Cambridge, UK; 5 Forget Me Not Children’s Hospice, Huddersfield, UK; 6 Neonatal Unit, Leeds Children’s Hospital, Leeds, UK; 7 University of Exeter, Exeter, UK; 8 Claire House Children’s Hospice, Liverpool, UK

## Abstract

There is a growing recognition that perinatal palliative care is appropriate whenever there is uncertainty about a baby’s survival outcome. Many aspects of this care can, and should, be provided by existing perinatal teams, with support from community and specialist services where required. However, delivering perinatal palliative care can be practically, ethically and emotionally challenging for professionals without adequate guidance and training. In this best practice paper, we offer practical guidance for clinicians who may be involved in antenatal counselling when a baby has been diagnosed with a potentially life-limiting condition during pregnancy. We consider how to approach antenatal counselling in the context of prognostic uncertainty, how to support families in being able to treasure their pregnancy, how to remain open to all possibilities, how to develop individualised care plans for families and finally consider how to close the consultation and arrange follow up. Drawing on the parental experience of one of our authors we explore how to navigate the concept of hope in perinatal consultations.

## Introduction

When a baby is diagnosed with a life-limiting or potentially life-limiting condition during pregnancy, families rely on healthcare professionals to provide accurate and compassionate counselling. Clinicians must balance medical information with sensitivity to parental emotions. This is especially challenging when prognoses are uncertain, and treatment options are evolving. Palliative care is increasingly recognised as an important component of this support in the antenatal period, allowing parents to prepare and plan for a range of possible outcomes [1,2]. Crucially, palliative care is not just appropriate for babies who will certainly die in early life. Integrating perinatal palliative care into antenatal care is appropriate whenever there is uncertainty about a baby’s (short or longer term) survival outcome. Such uncertainty about whether a baby will survive may arise from fetal, pregnancy-related or prematurity-related conditions [3].

Perinatal palliative care can be delivered in conjunction with specialist paediatric palliative care teams. However, many aspects of care can, and should, be provided by frontline perinatal and paediatric teams [4]. Approaching sensitive conversations about palliative care before birth can feel daunting for professionals. In this paper, we provide practical guidance on how to approach antenatal counselling in the context of a potentially life-limiting diagnosis.

### Counselling in uncertainty

When planning an antenatal consultation it is important that diagnostic results are delivered by someone who is confident and competent in sensitively disclosing what could be difficult news or complex information [5]. Counselling should be honest and clear and based on available current evidence with the aim of helping parents to understand the likelihood of different outcomes in their particular case. When using technical terms these should be written down together with their lay interpretations. It can be helpful to include a member of staff who has already met the family and to ensure continuity of staff throughout the pregnancy wherever possible.

A potential outcome of pregnancy with significant concerns about a baby’s health is termination for medical reasons (TFMR). This should be discussed in a non-directive, sensitive manner, taking into account the stage of the pregnancy and considering the impact on the physical and emotional health of the mother. All parents facing a potentially life-limiting diagnosis in pregnancy, including those considering a TFMR, may benefit from early involvement of paediatric services and relevant specialist teams, including palliative care. It is best practice to arrange joint consultations which involve fetal medicine alongside paediatric teams to avoid repetitive or conflicting messaging to families. Counselling should be parent led. It is therefore important to establish at the outset what parents hope to gain from the consultation, what questions or concerns they have, and how they wish to receive information. For example, some families will wish to discuss all eventualities in detail whereas others may favour a more stepwise approach to receiving information. This may also change throughout the pregnancy.

Value-laden language should be avoided (particularly the use of outdated phrases such as ‘incompatible with life’ or ‘lethal malformation’) and neutral terms such as ‘expected’ and ‘unexpected’ are preferable to ‘normal’ and ‘abnormal’ [6,7]. If a parent has given their baby a name, try to refer to the baby by their name in your discussions and correspondence.

Considerable uncertainty about a baby’s prognosis during the antenatal period is not uncommon. This may reflect the limitations of prenatal diagnostics, including where parents have declined antenatal testing, or the nature of the condition itself. Uncertainty can be distressing for both parents and professionals to navigate but it is important that uncertainty is communicated honestly to expectant parents and that a balanced picture of all possible outcomes is presented. Even when the prognosis is very guarded, many families will remain hopeful for a positive outcome and as professionals it is important to allow space for this hope to exist [8,9]. Box 1 offers a parental perspective on navigating hope in the perinatal period.

Box 1 **A parent’s perspective on navigating hope in the perinatal period**

Our daughter has a diagnosis of Edwards’ syndrome, and the only information we were given at her diagnosis was very bleak. I have often reflected on the impact of those difficult early conversations on our family, both in the neonatal period and throughout her childhood. I have noticed that some professionals would rather focus on the certainty of death, and that this drives the conversations towards helping to ensure a good death - rather than helping the family to work out what a good life might look like, even if this is likely to be very short. And the importance of hope – even if it is a different kind of hope, like the hope to meet your baby, the hope that your baby might get to come home with you, and perhaps that tiny bit of hope that your baby might do better than expected. Those little glimmers of hope are so important and help you to keep going – whilst you know the reality of the situation it is really hard when you feel someone is trying to crush any hope that you have. And those moments you have with your baby are so precious, especially as they are likely to be so short. So during pregnancy and when your baby is born (and from talking to other parents - even if the baby is not born alive) you don’t want to spend **all** the time mentally planning their funeral and thinking about a bleak future without your baby in it.

### Finding ways to treasure the pregnancy

**Figure 1:**
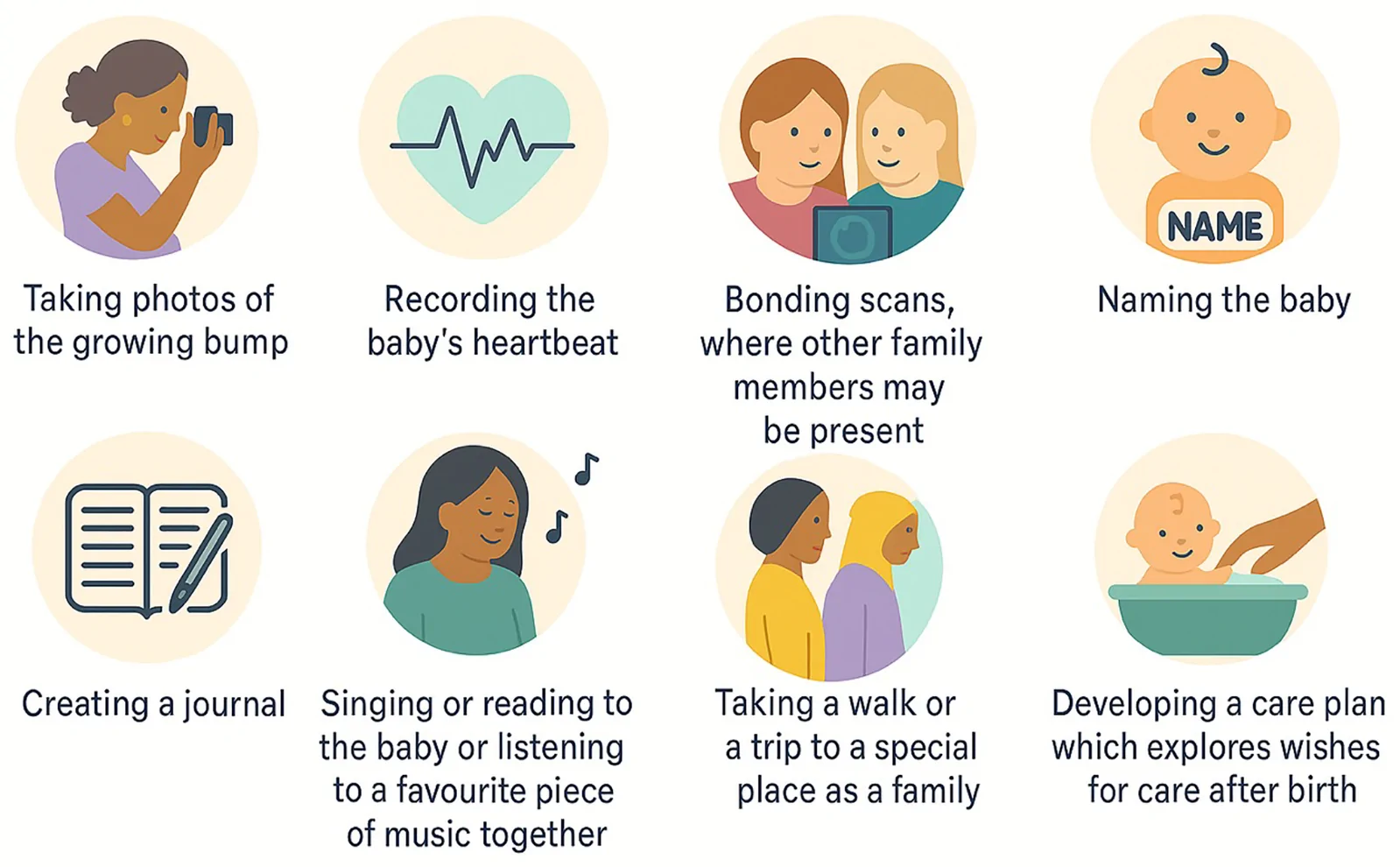
Suggestions for finding ways to treasure the pregnancy

Every pregnancy is unique, and families may experience a whole range of deep feelings and emotions which may change frequently over the course of the pregnancy. Bonding with the unborn baby can help families to honour the life of their baby and also their experience as a parent. There is no right way to go about this as the reality of the situation faced by families is deeply personal. Figure 1 includes some suggestions that can be shared with families.

There is a meaningful connection in every moment that parents have with their unborn baby, and it is important to validate the whole spectrum of parental emotions. The journey of a pregnancy that contains significant uncertainty can be deeply traumatic in and of itself and it is important to create a space where parents feel safe and free to ask questions or make requests without fear of judgement. Where there are siblings, it is important to discuss with parents how they would like them to be included, as well as what support they might need.

### Remaining open to all possibilities

Perhaps the most powerful approach a professional can take when approaching antenatal counselling in the context of a potentially life-limiting condition is to remain open to all possibilities. This requires us to avoid ‘black and white’ thinking and to consider each family and each baby as unique, rather than defining them by their diagnosis. Fetal medicine and obstetric teams should consider early involvement of paediatric and neonatal services. Palliative care teams should also be consulted whenever there is concern about a baby’s survival outcome and the extent of their involvement may vary throughout the pregnancy and neonatal period in response to clinical need. It is also important to explain that palliative care involves the holistic support of a child and their family and will often be provided alongside elements of survival-focused care. This may be particularly important where families have opted for medical interventions after birth as they may be worried that palliative care is synonymous with end-of-life care or an automatic cessation of treatment.

If a baby is diagnosed with a life-limiting or a life-threatening condition it is important to still actively consider what investigations and interventions may be useful or appropriate at birth, to either confirm or refine the diagnosis and to ensure the best possible treatment and outcome. This may involve consulting other paediatric specialist teams during the pregnancy such as neurosurgical or cardiology teams to ensure that care is optimised at birth. Consider early involvement of community services such as the local children’s community nursing (CCN) team and a children’s hospice during the antenatal period to maximise the support available to the family. Differences between parental views and those of the healthcare team may give rise to conflict. Seeking specialist opinions early, involving palliative care, or considering review by a clinical ethics committee can help to mitigate this risk [10].

### Developing a holistic individualised care plan

Parents should be offered the opportunity to develop a holistic, personalised care plan for their baby. Care planning should not be rushed, and ideally, conversations should take place over several face-to-face appointments. Some parents will not want, or feel able, to engage in care planning, or will only feel able to consider certain elements antenatally, and this should also be respected.

**Figure 2:**
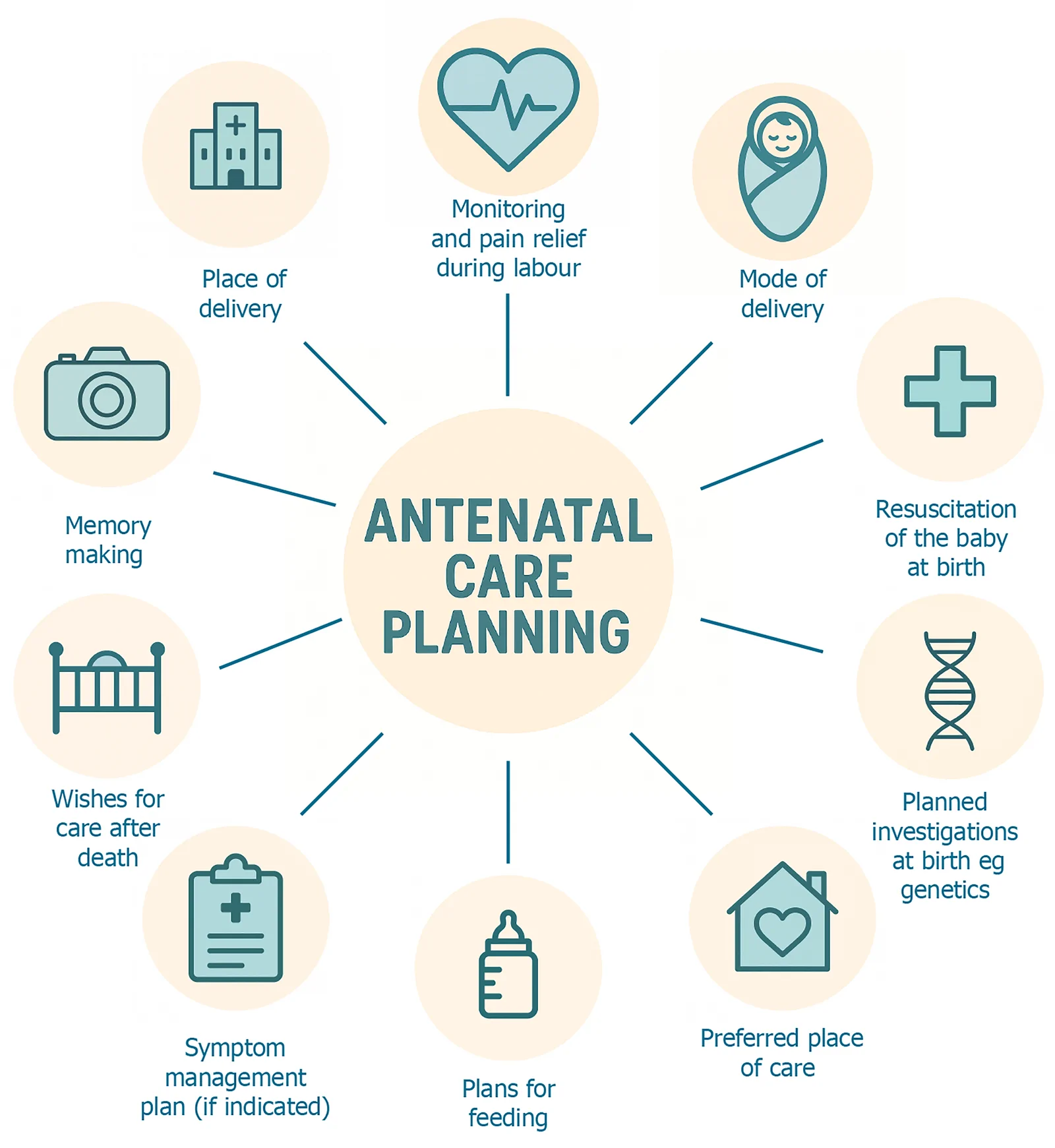
Suggested elements of antenatal care planning

Care planning should cover all aspects of the mother’s and baby’s care and may include a number of elements. It involves exploring all the potential outcomes for the baby and developing a plan for each of these. Figure 2 gives some examples of items the care plan may cover. Within these elements it will be important to discuss all the options that might be feasible, to discuss the potential risks and benefits of each option, and to give parents enough information in order to work with them to weigh up the alternatives.

It is also important to give parents the opportunity to explore their wishes for care after death, but also to respect that some parents may not want to talk about these whilst the baby is living. These may include whether they would like to take their baby to a different location after death, spiritual or religious customs, wishes around post-mortem, plans for a funeral, organ donation and milk donation or cessation.

Parents’ feelings and wishes should be central to care planning. Development of a care plan does not equate to a plan for end-of-life care or comfort care from birth. Many families will choose survival-focused interventions for their baby but wish to develop a plan in case these are not successful (“hoping for the best, planning for the worst”) e.g. for a baby with complex congenital heart disease. It is vital that parents feel that their baby matters. In some circumstances, it may be appropriate to offer a limited trial of interventions, such as non-invasive breathing support, even if this is unlikely to alter the overall trajectory of the baby’s condition. Box 2 details some of the more challenging questions which may come up during consultations and suggested ways of responding to these.

### Closing the consultation and follow up

When the consultation is coming to a close be sure to communicate what will happen next and when you plan to see the family again, as well as how they can contact the team if needed. Wherever possible offer a written summary of the consultation as well as any relevant written information (including diagrams) about their baby’s condition and signpost to relevant organisations which can offer further information and/or support (see Box 3 for some of the organisations you may wish to signpost to). Consider providing translated versions of written materials if English is not the family’s first language. It can be helpful to actively consider who will be providing bereavement support in the event of either a pregnancy loss or a neonatal death and to think carefully about both immediate and longer term support for the whole family [11,12].

Handling these sensitive conversations can be emotive and draining for professionals and it is important to consider what support is available for the staff involved in the consultation. Expanding palliative care education across the wider multidisciplinary team can help ensure that all staff are aware of the full range of options available to families. An online training course ‘Managing Uncertainty in Perinatal Medicine and Palliative Care’ is available for professionals via FutureLearn (www.futurelearn.com).

### Summary

Box 4 summarises the key clinical learning points for approaching antenatal counselling in perinatal palliative care.

Box 2 **Questions parents may ask during antenatal counselling for a potentially life-limiting condition**

**Will I be able to hold my baby?**

It is often helpful to explain exactly what is likely to happen after the baby is born, if the baby is likely to need to go to the neonatal unit and what to expect in terms of the baby’s appearance and symptoms. Part of this discussion will involve explaining the different possible management options for the baby, including whether some or full resuscitation will be offered, and where the baby will be when this takes place. Many parents may be worried about what their baby will look like and if they will be in pain. All parents should be given the opportunity to hold their baby as soon as possible, even if this is after a baby has died.

**What should we buy?**

Shopping for the nursery and choosing a pram is often one of the joyful milestones of pregnancy. However, for families facing uncertainty that excitement can be overshadowed by worry and grief. Many parents in this situation find themselves unsure about what they should or shouldn’t buy before the birth. Many families will want to pick out a baby blanket and some newborn clothes and these items may become important keepsakes.

For some, preparing the nursery, buying clothes or picking out a pram offers a sense of comfort and hope. For others, even the thought of shopping can feel overwhelming or painful. There is no right or wrong approach—what matters most is what feels right for each individual family. Everyone copes in their own way, and whatever choice is made, it deserves to be respected and supported.

**Can we have a funeral and where do we start to organise one?**

While having a funeral is a personal choice, every baby, no matter the stage of pregnancy or the circumstances of their birth, can be honoured with a funeral. Many parents find that, despite the sadness and pain it may bring, it offers a meaningful way to recognise and celebrate their baby’s brief but precious life. Not every family will choose to have a funeral but there is a legal requirement that a burial or cremation takes place, and the hospital can arrange this. If a baby is born dead after 24 completed weeks of pregnancy, or lived for a short time and then died, the baby’s stillbirth, or birth and death, must be registered by the local registrar. Before 24 weeks the death cannot be registered but a Medical Certificate from the hospital is required before a burial or cremation can take place. Sands charity have produced a guide ‘Deciding about a funeral for your baby’ [13] which provides clear and practical guidance on what needs to be done if the family decide to have a funeral, and the choices they can make.

**Is organ donation an option?**

Antenatal counselling about organ donation is a sensitive yet deeply meaningful conversation, particularly in pregnancies where the baby has a life-limiting condition. For some families, exploring the possibility of organ or tissue donation provides a way to create a lasting legacy for their baby—a way to honour their life by helping others.

Counselling offers expectant parents the chance to understand their options and make informed, value-based decisions. The first step is to contact your local Specialist Nurse in Organ Donation (SNOD). They will be able to assess medical eligibility based on individual circumstances and explain what types of donation may be possible. This includes specific criteria such as gestation, weight, and the timing of death after delivery. It is important to explain that neonatal organ donation in the UK is currently very rare. The process can be complex, and it is not always possible to proceed. Even if donation turns out not to be possible, the SNOD will speak directly with the family to explain the reasons clearly and with compassion. These conversations are handled with great care, and support is available throughout the process.

Box 3 **Sources of support for parents following an antenatal diagnosis of a potentially life-limiting condition in their baby**

- ANTENATAL RESULTS AND CHOICES (ARC) https://www.arc-uk.org/ Impartial information supporting parents through antenatal testing and its consequences.
- TOGETHER FOR SHORT LIVES https://www.togetherforshortlives.org.uk/
  UK’s leading charity for Children’s Palliative Care, provides signposting toward local services including children’s hospices.
- SOFT UK https://www.soft.org.uk/
  Support and information regarding Trisomy 13 and Trisomy 18.
- SANDS https://www.sands.org.uk/
  Support to anyone affected by the death of a baby, before, during or shortly after birth.
- FOOTPRINTS BABY LOSS https://footprintsbabyloss.org
  Support to parents and families who experience the death of one or more of their twins or triplets before, during or after birth.
- PERINATAL PALLIATIVE CARE https://www.perinatalpalliativecare.org/
  An information resource for families and professionals all about perinatal palliative care

Box 4 **Key clinical learning points**

- Palliative care can begin in the antenatal period and is appropriate in all cases where there is uncertainty about the baby’s (short or longer term) survival outcome
- If there is uncertainty about a baby’s prognosis, it is best to openly acknowledge this with families and to discuss the full range of potential outcomes
- The diagnosis does not define the child. Keep an open mind about what treatments or interventions may be possible and remember that each baby and family will be unique
- Developing an individualised, holistic antenatal care plan can help parents to realise their wishes for the care of their baby after birth
- Set aside sufficient time for the consultation. Antenatal counselling and care planning in perinatal palliative care takes time and many conversations will need to be revisited throughout the pregnancy
- Antenatal counselling can be emotionally draining for professionals, and thought should be given to ensuring that adequate staff support is in place

## References

[1] Sidgwick P, Harrop E, Kelly B (2017). Fifteen-minute consultation: perinatal palliative care. Arch Dis Child Educ Pract Ed.

[2] Bertaud S, Brightley G, Crowley N (2023). Specialist perinatal palliative care: a retrospective review of antenatal referrals to a children’s palliative care service over 14 years. BMC Palliat Care.

[3] Wilkinson D, Bertaud S, Mancini A (2025). Recognising uncertainty: an integrated framework for palliative care in perinatal medicine. Arch Dis Child Fetal Neonatal Ed.

[4] Harrop E, Edwards C (2013). How and when to refer a child for specialist paediatric palliative care. Arch Dis Child Educ Pract Ed.

[5] ARC (2024). Parent-centred guidelines for care after diagnosis of an NHS FASP condition.

[6] Wilkinson D, Thiele P, Watkins A (2012). Fatally flawed? A review and ethical analysis of lethal congenital malformations. BJOG.

[7] Johnson J, Areizina J, Tomlin L (2020). UK consensus guidelines for the delivery of unexpected news in obstetric ultrasound: The ASCKS framework. Ultrasound.

[8] Bertaud S, Suleman M, Wilkinson D (2025). Hope pluralism in antenatal palliative care. J Med Ethics.

[9] Pearson A (2015). Never say never about our child. BMJ.

[10] Nuffield Council on Bioethics (2023). Independent review: disagreements in the care of critically ill children.

[11] Johnston SE, McAllister S, Norden C (2024). Child bereavement-what matters to the families. Part 1: Immediate and short-term communication and care. Arch Dis Child Educ Pract Ed.

[12] Johnston SE, McAllister S, Norden C (2024). Child bereavement-what matters to the families. Part 2: The long term. Arch Dis Child Educ Pract Ed.

[13] Sands (2014). Deciding about a funeral for your baby.

